# High frequency of Human Cytomegalovirus DNA in the Liver of Infants with Extrahepatic Neonatal Cholestasis

**DOI:** 10.1186/1471-2334-5-108

**Published:** 2005-12-01

**Authors:** Adriana MA De Tommaso, Paula D Andrade, Sandra CB Costa, Cecília AF Escanhoela, Gabriel Hessel

**Affiliations:** 1 Department of Pediatrics, Faculty of Medical Sciences, State University of Campinas (UNICAMP), Campinas-SP, Brazil; 2 Department of Internal Medicine, Faculty of Medical Sciences, State University of Campinas (UNICAMP), Campinas-SP, Brazil; 3 Department of Pathology, Faculty of Medical Sciences, State University of Campinas (UNICAMP), Campinas-SP, Brazil

## Abstract

**Background:**

Biliary atresia (BA) is the most severe hepatic disorder in newborns and its etiopathogenesis remains unknown. Viral involvement has been proposed, including the human cytomegalovirus (HCMV). The aims of the study were to use the polymerase chain reaction (PCR) to screen the liver tissue of infants with extrahepatic cholestasis for HCMV and to correlate the results with serological antibodies against HCMV and histological findings.

**Methods:**

A retrospective study in a tertiary care setting included 35 patients (31 BA, 1 BA associated with a choledochal cyst, 2 congenital stenosis of the distal common bile duct and 1 hepatic cyst). HCMV serology was determined by ELISA. Liver and porta hepatis were examined histologically. Liver samples from infants and a control group were screened for HCMV DNA.

**Results:**

Twelve patients had HCMV negative serology, 9 were positive for IgG antibodies and 14 were positive for IgG and IgM. Nine liver and seven porta hepatis samples were positive for HCMV DNA but none of the control group were positive (general frequency of positivity was 34.3% – 12/35). There was no correlation between HCMV positivity by PCR and the histological findings. The accuracy of serology for detecting HCMV antibodies was low.

**Conclusion:**

These results indicate an elevated frequency of HCMV in pediatric patients with extrahepatic neonatal cholestasis. They also show the low accuracy of serological tests for detecting active HCMV infection and the lack of correlation between HCMV positivity by PCR and the histopathological changes.

## Background

The most common cause of extrahepatic neonatal cholestasis is biliary atresia (BA). BA is an obstructive cholangiopathy and the end result of a destructive inflammatory process that affects the biliary ductal system. This disease is more common in girls and involves 0.8–1/10000 live births [1]. The etiology of BA remains unknown but viral infections have been implicated with several viruses studied [2-12], including human cytomegalovirus (HCMV) [13-17]. The etiology of the other causes of extrahepatic neonatal cholestasis remains unknown.

HCMV is a slow replicating virus that belongs to the herpesvirus family (betaherpesvirus subfamily). Congenital infection by HCMV occurs in approximately 1% of all neonates. Of this 1%, only 5–10% have a typical cytomegalic inclusion disease with hepatosplenomegaly, jaundice and petechias. Another 10% will have a subclinical congenital infection [18], and the remaining 80–85% will be asymptomatic. Virus detection in the urine, saliva or tissues of neonates during the first three weeks of life defines congenital infection [19]. In contrast to infections such as rubella and congenital toxoplasmosis, maternal immunity against HCMV does not always provide adequate protection against intrauterine transmission [19]. Hepatic involvement is frequent and clinical evidence of hepatitis is occasionally found [20-22].

Perinatal infection is diagnosed in children who do not eliminate the virus in urine at birth but who begin to show the virus between the 4^th ^and 12^th ^week of life. The infection results from viral transmission during delivery, through maternal milk or by blood transfusions, the first two of these being the most important. The vast majority of infants remain asymptomatic [19].

The purpose of this study was to screen the liver tissue of infants with extrahepatic neonatal cholestasis for the presence of HCMV using PCR, and to correlate the results with the HCMV serologies (ELISA systems) and histopathological findings in these patients.

## Methods

Thirty-five patients (13 males and 22 females) with extrahepatic neonatal cholestasis were evaluated upon admission to the Pediatric Gastroenterology Service of the university hospital at UNICAMP, from September 1992 to July 2000. The median infant age at the time of the first visit was 82.5 days (range 25–239 days). Neonatal cholestasis was secondary to BA in 31 infants and BA was associated with a choledochal cyst in one, to distal choledochal stenosis in two, and to hepatic cyst (measuring 3.2 × 3.4 cm, close to the porta hepatis, compressing the biliary tree and leading to obstructive cholestasis) in one.

The diagnosis of BA was confirmed by intraoperative cholangiogram. The diagnosis of choledochal cyst was made by ultrasound and the distal choledochal stenosis was diagnosed by intraoperative cholangiogram.

Liver tissue samples of nine infants were included as controls and had presented the following diagnoses: 1 had drug-related hepatotoxicity, 2 had alpha-1-antitrypsin deficiency, 2 had galactosemia, 1 had cystic fibrosis, 1 had congenital hepatic fibrosis, 1 had hepatoblastoma and 1 had a metabolic disease of undefined cause. The median infant age was 46 days (range 28–180 days).

### Serological investigation

The patients were divided into three groups, based on the CMV serology results (by ELISA commercial system: SORIN BIOMEDICA, Italy): 1) ELISA IgG-/IgM-; 2). ELISA IgG+/IgM-, and 3) ELISA IgG+/IgM+. IgM antibodies were detected by ELISA-capture assay.

### Histological Analysis

All histological analyses were done by the same pathologist. Fifty three samples (33 liver tissues and 20 porta hepatis fragments) were evaluated by PCR. Of the 33 liver tissues evaluated, 10 were collected by percutaneous biopsies, while the others were surgical biopsies. The following characteristics were evaluated: portal fibrosis (0 = absent, 1 = slight, 2 = moderate, 3 = severe), septa (0 = absent, 1 = mild, 2 = moderate, 3 = severe), nodules (P = present, A = absent), cholestasis and cholangitis (0 = absent, 1 = mild, 2 = moderate, 3 = severe), giant cell transformation (P = present, A = absent), eosinophils (P = present, A = absent), myeloid metaplasia (P = present, A = absent), siderosis (0 = absent, I = degree I, II = degree II, III = degree III), cytomegalic inclusion (P = present, A = absent), microabscesses (P = present, A = absent), ductal proliferation (0 = absent, 1 = slight, 2 = moderate, 3 = intense), porta hepatis (normal, chronic inflammatory process with predominance of lymphomononuclear cells or chronically active with predominance of polymorphonuclear neutrophils or macrophages) and inflammatory process of the porta hepatis (1 = mild, 2 = moderate, 3 = severe).

### DNA extraction/amplification

DNA was extracted from fresh material (4 patients) according to Rogers *et al*. [23] and from formalin-fixed paraffin-embbeded fragments by phenol-chloroform procedure described by Latchman [24]. PCR conditions for HCMV and β-globin were the same. Five sections of 10 μm thick were obtained for DNA amplification [25]. The human β-globin gene was amplified according SAIKI *et al*. [26]. HCMV was detected by PCR and nested-PCR, according Saiki *et al*. [26], Shibata *et al*. [27] and Demmler *et al*. [28].

The amplifications were done in a DNA thermocycler (Robocycler 40 – Stratagene) using 35 cycles of 94°C for 45 s, 55°C for 45 s and 72°C for 1 min. The cycles were preceded by an initial denaturation at 94°C for 5 min and were followed by a final extension for 7 min at 72°C. The primers for the human β-globin gene were: PCO3+ (5' CCTCTGACACAACTGTGTTCACTAGC 3') and PCO4+ (5' TCACCACCAACTTCATCCACGTTCACC 3'). The primers for HCMV DNA were: MIE4 (CCA AGC GGC CTC TGA TAA CCA AGC C), MIE5 (CAG CAC CAT CCT CCT CTT CCT CTG G), IE1 (CCA CCC GTG GTG CCA GCT CC) and IE2 (CCC GCT CCT CCT GAG CAC CC). The MIE4 and MIE5 primers were used for the first reaction (PCR) and IE1 and IE2 for the nested-PCR.

The reaction mixture consisted of 0.5 – 1.0 μl of DNA in a total volume of 20 μl containing 50 mM KCl, 10 mM Tris (pH 8.4), 3 mM MgCl, 0.1 mM of each primer, 200 mM of each deoxyribonucleotide triphosphate (dATP, dGTP, dCTP, dTTP) and 2–4 units Taq polymerase. Water was used to complete the total reaction volume. The mixture was covered with a drop of mineral oil.

The reaction products were separeted by electrophoresis in 2% agarose gels, stained with ethidium bromide and visualized under ultraviolet light. For each CMV reaction, fibroblast fluid containing strain AD 169 was used as a positive control and the reaction mixture without the strain was used as a negative control. Recommended procedures were used to avoid contamination [23].

The statistical analysis consisted of a descriptive analysis with a frequency table, Fisher's exact test, the Kappa coefficient and accuracy measurements. The significance level was set at 5%. This study was approved by the ethics committee of the Faculty of Medical Sciences, UNICAMP, and all infant's parents provided their informed consent prior to enrolling in the study.

## Results

Active HCMV infection was defined if one or both of the following conditions are present: positive result for HCMV DNA by PCR and a positive test for IgM HCMV antibodies.

Twelve patients had HCMV negative serology (group I), 9 were positive for IgG antibodies (group II) and 14 were positive for IgG and IgM (group III). In group I, 11 patients had BA (associated with a *situs inversus *in one patient) and one patient had a distal choledochal stenosis. All of the patients in group II had BA and in group III, 12 children had BA (associated with a choledochal cyst in one of them), one had a distal choledochal stenosis and another had a hepatic cyst. The general characteristics of the patients are show in Table 1. All were full term neonates. The median time between HCMV serology and liver biopsy was 8 days.

**Table 1:** Gender and median of birth weigh, alanine aminotransferase (ALT), gamma-glutamyl transpeptidase (GGT) and direct bilirubin (DB) in the patients.

CHARACTERISTICS/DIAGNOSIS	GENDER	BIRTH WEIGH	ALTi	GGTi	DBi (mg%)
BA* (10)	5F/5M	3180 g	4	12.2	22.7
Hepatic cyst* (1)	F	2970 g	2.9	3.5	19.7
Stenosis of the distal common bile duct *(1)	F	?	6.6	5.4	31
BA (21)	13F/8M	2950 g	5.3	16	23.7
BA + Choledocal cyst (1)	F	3100 g	2.5	27.5	15.2
Stenosis of the distal common bile duct (1)	F	3100 g	2.3	11.8	11.7

**Figure 1:**
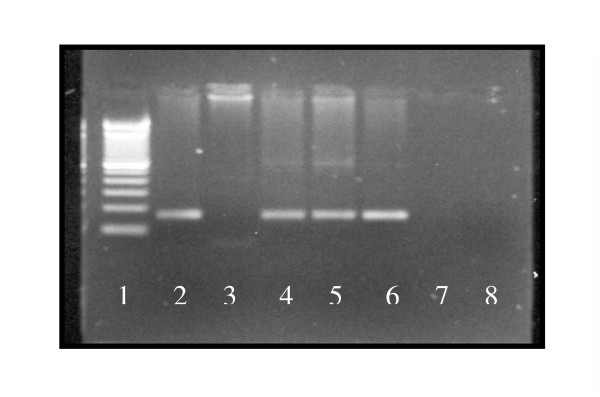
HCMV DNA results in some patients. 1 = Ladder 2 = Positive control 3 = Porta-hepatis fragment from patient A (CMV-) 4 = Liver tissue from patient A (CMV+) 5 = Liver tissue from patient B (CMV+) 6 = Urine from patient B (CMV+) 7 = Empty lane 8 = Negative control

* = pcr positive patients, M = male; F = female; ALTi = ALT divided by the upper reference value; GGTi = GGT divided by the upper reference value; DBi = DB divided by the upper reference value;

Of 33 liver samples screened by PCR, 9 (27.3%) were positive for HCMV DNA and 7 out of 20 (35%) porta hepatis fragments were positive (figure 1). There was adequate agreement between these results (Kappa coefficient = 0.51), albeit some divergences. Two patients had HCMV DNA in the liver but not in porta hepatic. Three children were positive for HCMV DNA in the porta hepatis but negative for HCMV in the liver tissue. The overall frequency of HCMV DNA was 34.3% (12/35) based on a positive result in one of the two tissue samples. Based on the PCR distribution among the groups there were three positive patients in group I, three in group II and six in group III.

The histological characteristics of HCMV positive patients are show in Table 2 and of HCMV negative patients in Table 3. The pathologist did not observe the presence of cytomegalic cells in any case. No significant correlation was observed between any of the findings.

**Table 2:** Histological characteristics of HCMV positive patients.

Diagnosis	PHF	Liver tissue							
		
		PF	GCT	Ductal proliferation	Septs	Nodules	Cholestasis	Cholangitis	Eosinophils
BA	MoCIP	+	+	+	+	+	+	+	+
BA^#^	no surgery	+	-	+	+	+	+	-	-
BA	normal	+	+	+	+	-	+	-	+
BA	MoCAP	+	+	+	+	-	+	+	+
BA	MCIP	+	+	+	+	+	+	+	+
BA	MCIP	+	+	+	+	+	+	+	+
BA	MCIP	+	+	+	+	+	+	+	+
BA	?	?	?	?	?	?	?	?	?
BA	MCAP	+	-	+	+	+	+	-	-
BA	normal	+	+	+	+	-	+	-	-
DCS	No	+	-	+	+	+	+	+	+
HC	No	+	+	+	+	+	+	-	+

BA = biliary atresia, DCS = distal choledocal stenosis, HC = hepatis cyst, PHF = porta hepatis fragment, # = *situs inversus *PF = portal fibrosis, GCT = giant cell transformation, MCIP = mild chronic inflammatory process, MoCIP = moderate chronic inflammatory process, MCPA = mild chronic active inflammatory process, MoCAP = moderate chronic active inflammatory process.

**Table 3:** Histological characteristics of HCMV negative patients.

Diagnosis	PHF	Liver tissue							
		
		PF	GCT	Ductal proliferation	Septs	Nodules	Cholestasis	Cholangitis	Eosinophils
BA	MoCAP	+	+	+	+	+	+	-	+
BA	SCAP	+	+	+	+	+	+	-	-
BA	MoCAP	+	+	+	+	-	+	-	-
BA	no surgery	+	-	+	+	-	+	-	-
BA	MCAP	+	+	+	+	+	+	-	+
BA	MCIP	+	+	+	+	+	+	-	+
BA	no surgery	+	-	+	+	+	+	-	+
BA	MCIP	+	+	+	+	-	+	+	-
BA	SCAP	+	+	-	-	-	+	-	-
BA	MCAP	+	+	+	+	-	+	+	+
BA	no surgery	+	-	+	-	-	+	-	-
BA	MoCAP	+	+	+	+	-	+	-	+
BA	MoCIP	+	+	+	+	+	+	-	+
BA	no surgery	+	+	+	+	+	-	+	
BA	no surgery	+	+	+	+	+	+	-	+
BA	MoCAP	+	+	+	+	+	+	-	+
BA	MoCAP	+	+	+	+	+	+	-	+
BA	MoCAP	+	-	+	+	-	+	-	+
BA	no surgery	+	+	+	+	+	+	-	+
BA	?	+	-	+	+	+	+	-	-
BA+CC	MoCAP	+	-	+	+	+	+	-	+
CC	MoCIP	+	+	+	+	+	+	+	+
DCS	No	+	-	+	+	+	+	_	+

BA = biliary atresia, DCS = distal choledocal stenosis, CC = choledocal cyst, PHF = porta hepatis fragment, PF = portal fibrosis, GCT = giant cell transformation, MCIP = mild chronic inflammatory process, MoCIP = moderate chronic inflammatory process, MCAP = mild chronic active inflammatory process, MoCAP = moderate chronic active inflammatory process, SCAP = severe chronic active inflammatory process.

Compared to PCR, serology had a low accuracy (59%) to detect active HCMV infection, with 54% sensitivity and 61% specificity. The patients' specimens from the control group had no HCMV DNA detected by PCR analysis. The table 4 shows the results of serology and PCR in all patients.

**Table 4:** Results of serology and PCR for detecting HCMV.

PATIENT	SEROLOGY	PCR – LIVER	PCR – PORTA HEPATIS
AFS	IgM-/IgG+	POSITIVE	POSITIVE
ADS	NEGATIVE	POSITIVE	NR
ADPS	IgM+/IgG+	NEGATIVE	NEGATIVE
AHL	IgM+/IgG+	NR	NEGATIVE
AGP	NEGATIVE	NEGATIVE	NEGATIVE
ACHA	NEGATIVE	NEGATIVE	NR
ACNG	IgM+/IgG+	NEGATIVE	NR
CT	IgM+/IgG+	POSITIVE	NR
CAAM	IgM+/IgG+	NEGATIVE	NR
CCA	NEGATIVE	POSITIVE	POSITIVE
DAMG	NEGATIVE	NEGATIVE	NR
DRA	IgM+/IgG+	NEGATIVE	POSITIVE
ESC	IgM-/IgG+	NEGATIVE	NEGATIVE
FRNP	NEGATIVE	NEGATIVE	NEGATIVE
GDS	IgM+/IgG+	NEGATIVE	NR
GFO	IgM-/IgG+	POSITIVE	POSITIVE
JG	NEGATIVE	NEGATIVE	NEGATIVE
JSO	IgM-/IgG+	NEGATIVE	NEGATIVE
LaGS	IgM-/IgG+	POSITIVE	NEGATIVE
LiGS	NEGATIVE	NEGATIVE	NEGATIVE
LS	IgM-/IgG+	NEGATIVE	NR
MCC	IgM+/IgG+	NEGATIVE	NEGATIVE
MFRF	NEGATIVE	NEGATIVE	NR
MFV	IgM+/IgG+	POSITIVE	NEGATIVE
NFAM	IgM-/IgG+	NEGATIVE	NR
PAL	IgM+/IgG+	POSITIVE	NR
RCM	IgM+/IgG+	NEGATIVE	POSITIVE
RRF	IgM-/IgG+	NEGATIVE	NR
RRX	IgM-/IgG+	NEGATIVE	NR
SCF	IgM+/IgG+	NR	POSITIVE
TDF	NEGATIVE	POSITIVE	POSITIVE
TFMF	IgM+/IgG+	NEGATIVE	NEGATIVE
YSR	NEGATIVE	NEGATIVE	NEGATIVE
WROS	IgM+/IgG+	NEGATIVE	NR
WGSS	NEGATIVE	NEGATIVE	NR

NR = no results

## Discussion

Progress has been made in the diagnosis, treatment and prognosis of BA and other causes of extrahepatic cholestasis. The etiopathogenic origin of the disease still remains unknown, although viral involvement has been suggested. Based on several diagnostic methods, including PCR, cytomegalovirus has been found in association with BA [13,14,16,17]. However, other studies have found no such correlation [12,15]. Of the other causes of obstructive neonatal cholestasis, only choledochal cysts have been associated with viral infections.

Sokol *et al*. [10] found no positivity for reovirus type 3 (29 patients with BA, 1 with a choledochal cyst) and Tyler *et al*. [15] found positivity in 7 of 9 children with choledochal cysts. Our study is the first to investigate the ocurrence of HCMV in other causes of extrahepatic neonatal cholestasis. HCMV DNA was found in patients with a hepatic cyst and a distal choledochal stenosis.

Serological screening revealed a seroprevalence of HCMV infection of 65.7% [23/35]. Previous studies made in Brazil have evaluated the incidence of congenital infection and prevalence of antibodies in mothers. Pannuti *et al*. [29] showed a prevalence of HCMV antibodies in mothers of 84.4% and the incidence of congenital infection was 0.98% in the low socioeconomic population. In the middle socioeconomic cohort, the maternal prevalence and incidence of congenital infections were 66.5% and 0.39%, respectively [29]. Yamamoto *et al*. [30] showed a prevalence of congenital infection of 2.6% with maternal seropositivity of 95%. On the other hand, the acquisition risk of perinatal infection was 30.9% [31]. Unfortunately, we don't have data regarding the serologic pattern of our patient's mothers since this test is not part of our institution's routine clinical practice.

The accuracy of serology to detected active HCMV infection compared with PCR in this study was low. The correlation between serology and PCR results suggests the need for caution in concluding, based on positive serology, that HCMV is responsible for the observed cholestasis. When the 14 patients with positive IgM serology were analyzed by PCR in biopsies, only 6 were positive. Thus, although 8 patients had an active HCMV infection detected by serology, the cholestasis was not secondary to this infection and its cause requires further investigation.

In several cases, a positive serology delayed referral of the patient to our service since they were considered to have hepatitis secondary to viral infection. This is important since most pediatricians begin the investigation of cholestasis by determining that infectious agents are involved, and suspend further analysis when a positive serology is found. Another factor to be considered in patients with positive IgM serology and negative PCR is the possibility of a false-positive result for IgM. False-positives can be secondary to the presence of rheumatoid factor (IgM of the neonate against maternal IgG), or to cross-reaction with other herpesviruses [22]. Until the screening of IgM antibody is improved, physicians should use more than one method in their diagnosis [22].

Of the 9 IgG+/IgM- patients, 3 were positive for HCMV DNA. The presence of IgG may represent the transplacental passage of maternal IgG (the infant being infected or not). With such a serological profile, the physician should be concerned not only with completing the investigation, but also with repeating the serology because of the possibility of IgM appearing in a subsequent collection, especially when the infant is infected. Another possibility would be to measure IgG levels as well, since they remain high in congenital or perinatal infection [32]. In patients with a positive PCR, the absence of IgM antibodies could represent a false-negative result. False-negative results may be secondary to the competition between the high levels of maternal IgG antibodies and the relatively low levels of fetal IgM [22]. In the post-natal period, when facing an antigenic stimulus, IgM levels rise rapidly during the first month of life and increase gradually. At around one year of age, these values reach approximately 60% of the adult levels [33].

Of the 12 ELISA-negative patients, 3 were positive by PCR. Sample contamination cannot be excluded, despite the low probability of this occurring in view of the care taken during extraction and PCR, including the use of nested PCR.

There was good agreement between liver and porta hepatis tissues when analysed separately. However, two patients with HCMV DNA in liver tissue were negative in the porta hepatis. In both, the band representing β-globin DNA in the porta hepatis fragment was considered weakly positive, while the corresponding hepatic band was considered strongly positive. This may indicate a false-negative result, judging by the sample DNA quality. Two patients were positive for HCMV DNA in the porta hepatis and negative in the liver. They had a positive serology (IgG+/IgM+) and very intense β-globin band. Since the HCMV was not genotyped, we cannot exclude the possibility of the existence of strains with different affinity for ducts or hepatocytes, and of strains with differences in their virulence. Further studies are needed to clarify this question.

In the absence of *in situ *hybridization it is unclear whether the detected HCMV was within the hepatocytes and/or biliary duct cells, or whether it was simply circulating in the plasma or in leucocytes.

In this study the pathologist did not observed the presence of cytomegalic cells or microabscess in any case. Similar results have been reported by others [9,14,16].

The 34.3% positivity for HCMV DNA found in infants with extrahepatic cholestasis contrast with the lack of positivity in any of the controls. These findings, and those of Fischler *et al*. [13] and Domiati-Saad *et al*. [11], indicate that cytomegalovirus may be involved in the etiopathogenesis of BA. This hypothesis could be tested with an experimental model of BA induced by HCMV in animals using the methodology described by Petersen *et al*. [34].

In contrast, Chang *et al*. [9] and Jevon *et al*. [14] found no HCMV involvement in the etiopathogenesis of BA. In the study by Jevon *et al*. [14], no HCMV was found in 12 infants with BA. A probable explanation for this finding could be the low prevalence of the infection in a Canadian population, which would not exclude viral participation in cholestasis. Chang *et al*. [9] examined the occurrence of HCMV in 50 infants with neonatal hepatitis (26 with BA) and 30 controls, and found positivity in two cases with BA, in 23 with hepatitis, but in none of the controls. A possible explanation for this finding could be the strain of virus involved.

The hypothesis that biliary epithelial cells function as antigen presenters was considered. In normal liver, MHC (major histocompatibility complex) class I antigens are expressed on the sinusoidal cells, large vessel and sinusoidal endothelium, biliary epithelium, and dendritic cells. Class II antigens are found only on the sinusoidal cells, capillary endothelium and dendritic cells. The ductal epithelium of BA patients has an aberrant expression of class II antigens [35]. Recently, specific morphological alterations in the biliary ducts has been described [36,37]. Injury to the epithelium secondary to an immune modulated response may be triggered by a viral infection.

If a viral infection contributes to this injury, then antiviral therapy in the infants with HCMV infection can be considered as a possible intervention and consequently may improve outcomes. Further controlled randomized studies are needed to clarify this aspect.

## Conclusion

In conclusion, the frequency of HCMV detected by PCR was high (34.3%). The accuracy of serology for detecting active HCMV infection was low compared to PCR, and there was no correlation between the presence of HCMV and the typical histopathological alterations (microabscess and cytomegalic cells) or the inflammatory process in porta hepatis in our patients.

## Authors' contributions

AMADT had primary responsibility for protocol development, patient screening, enrolment, outcome assessment, laboratory investigation, preliminary data analysis and writing the manuscript.

PDA participated in the development of the protocol, laboratory investigation and contribut to the writing of the manuscript.

SCBC participated in the development of the protocol and analytic framework for the study, and contribut to the writing of the manuscript.

CAFE was responsible for histological analysis and contributed to the writing of the manuscript.

GH supervised the design and execution of the study, performed the final data analysis and contributed to the writing of the manuscript.

All authors read and approved the final manuscript.

## Pre-publication history

The pre-publication history for this paper can be accessed here:


